# Altered Expression of Immune-Related Genes in Children with Down Syndrome

**DOI:** 10.1371/journal.pone.0107218

**Published:** 2014-09-15

**Authors:** Bruna Lancia Zampieri, Joice Matos Biselli-Périco, Jorge Estefano Santana de Souza, Matheus Carvalho Bürger, Wilson Araújo Silva Júnior, Eny Maria Goloni-Bertollo, Érika Cristina Pavarino

**Affiliations:** 1 Unidade de Pesquisa em Genética e Biologia Molecular, Departamento de Biologia Molecular, Faculdade de Medicina de São José do Rio Preto (FAMERP), São José do Rio Preto, São Paulo, Brazil; 2 Department of Genetics and, Ribeirão Preto Medical School University of São Paulo, São Paulo, Brazil; 3 Institute of Bioinformatics and Biotechnology (2bio), São Paulo, Brazil; 4 National Institute of Science and Technology in Stem Cell and Cell Therapy and Center For Cell Based Therapy, Ribeirao Preto, São Paulo, Brazil; The Walter and Eliza Hall of Medical Research, Australia

## Abstract

Individuals with Down syndrome (DS) have a high incidence of immunological alterations with increased susceptibility to bacterial and viral infections and high frequency of different types of hematologic malignancies and autoimmune disorders. In the current study, we profiled the expression pattern of 92 immune-related genes in peripheral blood mononuclear cells (PBMCs) of two different groups, children with DS and control children, to identify differentially expressed genes that might be of pathogenetic importance for the development and phenotype of the immunological alterations observed in individuals with DS. PBMCs samples were obtained from six DS individuals with karyotypically confirmed full trisomy 21 and six healthy control individuals (ages 2–6 years). Gene expression was profiled in duplicate according to the manufacturer's instructions provided by commercially available TaqMan Human Immune Array representing 92 immune function genes and four reference genes on a 96-plex gene card. A set of 17 differentially expressed genes, not located on chromosome 21 (HSA21), involved in immune and inflammatory pathways was identified including 13 genes (*BCL2*, *CCL3*, *CCR7*, *CD19*, *CD28*, *CD40, CD40LG*, *CD80*, *EDN1*, *IKBKB*, *IL6, NOS2* and *SKI*) significantly down-regulated and four genes (*BCL2L1*, *CCR2*, *CCR5* and *IL10*) significantly up-regulated in children with DS. These findings highlight a list of candidate genes for further investigation into the molecular mechanism underlying DS pathology and reinforce the secondary effects of the presence of a third copy of HSA21.

## Introduction

Down syndrome (DS) is the most common chromosomal disorder and is associated with many medical conditions. Individuals with DS have a high incidence of immunological alterations with increased susceptibility to bacterial and viral infections and high frequency of different types of hematologic malignancies and autoimmune disorders. Leukemia, respiratory tract infections and autoimmune phenomena such as acquired hypothyroidism, celiac disease and diabetes mellitus, occur at higher frequency compared with non-DS individuals [1]–[4]. Even though improved medical care and progress in treatment, including the development of vaccines and antibiotics, have led to increased life expectancies of individuals with DS, they continue to have major health concerns associated with immunological impairment [5], [6].

Although differences between the immune system of children with DS and that of the general population have been described and investigated over the years, the etiologic basis of the immunological alterations observed in these individuals remains unclear. A number of gene expression studies using a variety of methods, including microarrays, quantitative real-time PCR (qPCR) and serial analysis of gene expression (SAGE) and a variety of human DS tissues have been conducted [7]–[11] but very few attempted to investigate a selected network associated to a specific DS feature such as immune impairment. Considering that the activation of immune system is reflected in changes in gene expression profiles of immune competent blood cells, and these changes are detectable in peripheral blood [12], [13], in the present study, we performed gene expression profiling of 92 immune-related genes in peripheral blood of children with DS and in control children, aiming to highlight a list of candidate genes and pathways associated with the immune abnormalities characteristically seen in DS individuals. As result, the comparison of gene expression in children with DS and controls showed 17 differentially expressed genes.

## Materials and Methods

### Ethics Statement

The study was approved by the Research Ethics Committee of São José do Rio Preto Medical School (CEP-FAMERP), State of São Paulo. Written informed consent was obtained from the parents or legal guardians of all study participants.

### Samples and groups

Blood samples drawn into EDTA collection tubes were obtained from six DS children with karyotypically confirmed full trisomy 21 (three male and three females) with a mean age of 4.00±1.41 years and 6 healthy control children (two male and four females) with a mean age of 3.82±1.09 years. Subjects that had personal history of chronic infection such as bronchitis, asthma and recurrent pneumonia, or clinical manifestations suggestive of acute infection including the flu, cough, fever and/or were under antibiotic treatment within 10 days preceding blood drawing did not meet the inclusion criteria for the study. Children with DS enrolled were attending routine follow-up at Genetics and Down syndrome Outpatient Pediatric Clinic at Hospital de Base de São José do Rio Preto (HB). Blood samples of control children were obtained from children attending routine follow-up at Outpatient Pediatric Clinic at HB and children from the day care center at Instituto de Biociências, Letras e Ciências Exatas - Universidade Estadual Paulista “Júlio de Mesquita Filho” (IBILCE-UNESP). During blood drawn an extra small amount of blood was collected for blood hemogram profiling.

### Total RNA isolation

Peripheral blood mononuclear cells (PBMCs) were freshly isolated by Ficoll Paque Plus (Sigma-Aldrich, St Louis, Missouri, USA) density gradient centrifugation of venous blood. Total RNA was extracted using TRIzol reagent (Invitrogen, Carlsbad, California, USA) following the manufacturer's protocol and RNA purity (A260/280 nm) was assessed using NanoDrop ND-1000 (NanoDrop Technologies, Wilmington, Delaware, USA). RNA integrity was evaluated by an Agilent 2100 Bioanalyzer (Agilent Technologies, Foster City, California, USA) and only samples with preserved 18S and 28S peaks and RNA integrity number (RIN) values equal or greater than 5 were selected for gene expression analysis.

### Quantitative real-time PCR

First, cDNA samples were prepared from total RNA samples using the High Capacity cDNA Reverse Transcription kit (Applied Biosystems, Carlsbad, California, USA). For each sample qPCR reactions were carried out in duplicate using 6.25 ng of cDNA and a 96-plex gene card, TaqMan Array Human Immune, Fast 96-Well Plate (Applied Biosystems, Carlsbad, California, USA), representing 92 immune-related genes and four reference genes (Table S1) according to the manufacturer's instructions. These reactions were performed using a StepOnePlus Real-Time PCR System (Applied Biosystems, Carlsbad, California, USA).

### Statistical analysis

A chi-square analysis was performed between the groups for gender comparison. For age and hemogram analysis, data characterized by a normal distribution were expressed as mean ± standard deviation and parameters without such distribution were expressed as median with range. Student's *t* test (normally distributed data) or the Mann-Whitney test (non-normally distributed data) was used for comparing the two groups. These statistical analyses were performed using the Minitab for Windows (Release 14) program and p-values less than 0.05 were considered statistically significant. Regarding the gene expression, normalization and fold change were calculated with the ΔΔCq method standardized by the geometric averaging of the expression level of the four reference genes [14], [15]. Genes with Cq value (quantification cycle, representing the cycle number in which the PCR products reach the threshold) equal 36 or lower were considered as detected. The relative gene expression levels were calculated based on the 2^−ΔΔCq^ method [14]. All expression analyses were performed in R statistical environment [16] using packages freely available under Bioconductor project [17]. The package HTqPCR [18] was used to preprocess the data and as an interface to other packages using as input the PCR data. The identification of differentially expressed genes was addressed using linear models for microarray data (LIMMA) [19]. In order to reduce the risk of false-positive, p-values were adjusted for multiple testing issues using Benjamini and Hochberg's method to control the false discovery rate (FDR) [20] and a gene was considered differentially expressed when the corresponding adjusted p-value was less than 0.05. Data from genes expressed in 50% or less of the samples in each group did not fit our criteria (i.e. be detected in at least four samples of each group) to be considered for statistical analyses.

To explore the degree of overlap between the dysregulated genes in our study with other gene expression studies, we downloaded available GEO datasets (http://www.ncbi.nlm.nih.gov/geo) and supplementary data of the 17 differentially expressed genes observed in our study from three of the latest gene expression profiling studies in DS: Lockstone et al. [8] (human adult DS brain), Li et al. [9] (PBMC) and Letourneau et al. [11] (fetal fibroblasts). For each gene, log_2_ expression fold change (log_2_[FC]) was determined by calculating the ratio of normalized expression values between the trisomic and the euploid samples (that is, a positive log_2_[FC] reflects the up-regualtion of the gene in the trisomic sample and a negative log_2_[FC] the down-regulation, with a 1.5 fold-change cut-off).

## Results

Age and sex distribution were similar among the groups indicating the homogeneity of these data in the total sample (P≥0.05). Hemogram analysis demonstrated that individuals with DS have significantly lower absolute count of total typical lymphocyte (P<0.0001) and leukocytes (P = 0.002) cells compared with control (Table 1).

**Table 1:** Absolute count of immune cells in children with Down syndrome (DS) compared with controls.

	DS individuals	Control individuals	P value
Leukocytes	5,483±1,535	9,172±1,317	0.002^a^
Neutrophils	2,734±792	3,240±796	0.298^a^
Basophils	32.73±9.30	31.60±22.4	0.909^a^
Typical lymphocytes	2,128±931	4,912±796	<0.0001^a^
Monocytes	468±121	590±297	0.388^a^
Eosinophils	109 (62.2–187.0)	221 (90.0–926.0)	0.1282^b^

a Data are expressed as mean ± standard deviation, and two-tailed t test was used for comparisons.

b Data is expressed as median (interquartile range), and Mann-Whitney test was used.

Comparison of gene expression in children with DS and controls resulted in 17 differentially expressed genes that met threshold for significance after correction for multiple testing. Four of them, BCL2-like 1 (*BCL2L1*), chemokine (C-C motif) receptor 2 (*CCR2*), chemokine (C-C motif) receptor 5 (gene/pseudogene) (*CCR5*) and interleukin 10 (*IL10*), were up-regulated, while 13 genes, B-cell CLL/lymphoma 2 (*BCL2*), chemokine (C-C motif) ligand 3 (*CCL3*), chemokine (C-C motif) receptor 7 (*CCR7*), CD19 molecule (*CD19*), CD28 molecule (*CD28*), CD40 molecule, TNF receptor superfamily member 5 (*CD40*), CD40 ligand (*CD40LG*), CD80 molecule (*CD80*), endothelin 1 (*EDN1*), inhibitor of kappa light polypeptide gene enhancer in B-cells, kinase beta (*IKBKB*), interleukin 6 (interferon, beta 2) (*IL6*), nitric oxide synthase 2, inducible (*NOS2*) and v-ski sarcoma viral oncogene homolog (avian) (*SKI*), were down-regulated (Table 2). Regarding the comparison between our data and that from Lockstone et al. [8], Li et al. [9] and Letourneau et al. [11] little overlap was observed (Table 3).

**Table 2:** Immune-related genes differentially expressed in peripheral blood mononuclear cells of individuals with Down syndrome compared with controls.

Gene	Description	Location	FC	P-value*	Important biological processes	Reference
*BCL2*	B-cell CLL/lymphoma 2	18	0.53062	0.00051	Survival of mature lymphocytes and memory B cells	[28]
*BCL2L1*	BCL2-like 1	20	1.44879	0.00588	Survival of immature lymphocytes	[35]
*CCL3*	Chemokine (C-C motif) ligand 3	17	0.30759	0.00084	Chemotaxis induction of immune cells; T-helper cells differentiation; eosinophil degranulation induction; histamine release from mast cells	[36]
*CCR2*	Chemokine (C-C motif) receptor 2	3	2.49185	0.00034	Monocyte chemotaxis mediation	[37]
*CCR5*	Chemokine (C-C motif) receptor 5 (gene/pseudogene)	3	1.35093	0.04714	Regulation of trafficking and effector functions of memory/effector T cells, macrophages and immature dendritic cells	[38]
*CCR7*	Chemokine (C-C motif) receptor 7	17	0.43481	0.01015	Thymocytes generation; central and peripheral tolerance; regulatory T cell function; T cell homeostasis	[39]
*CD19*	CD19 molecule	16	0.50585	0.04625	Modulation of signal transduction through B cell antigen–receptor complex; peripheral B cells activation and proliferation	[40]
*CD28*	CD28 molecule	2	0.58229	0.02596	Homeostasis of regulatory T cells (CD28/CD80); activation and optimal function of T cells; peripheral tolerance	[41], [42]
*CD40*	CD40 molecule, TNF receptor superfamily member 5	20	0.73739	0.04977	T cell-dependent immunoglobulin class switching; memory B cell development; germinal center formation	[43]
*CD40LG*	CD40 ligand	X	0.52433	0.00650	Regulation of B cell function	[43]
*CD80*	CD80 molecule	3	0.52103	0.00767	T cell proliferation; cytokine production	[44]
*EDN1*	Endothelin 1	6	0.29025	0.00319	Macrophages activation	[45]–[47]
*IKBKB*	Inhibitor of kappa light polypeptide gene enhancer in B-cells, kinase beta	8	0.61495	0.01943	Activation of the NFKB pathway	[48]
*IL6*	Interleukin 6	7	0.30417	0.00128	Antibody and autoantibody production; T cells activation; B cell differentiation; hematopoiesis	[49]
*IL10*	Interleukin 10	1	2.00380	0.00767	Secretion of cytokines like IL6, IL2 and interferon, gamma (IFNG); macrophages and B and T cells activation, differentiation and proliferation	[50]
*NOS2*	Nitric oxide synthase 2, inducible	17	0.45730	0.04625	Nitric oxide synthesis (microbicidal, antiviral, antiparasital and antitumoral effects)	[51]
*SKI*	V-ski sarcoma viral oncogene homolog (avian)	1	0.72524	0.04977	Regulation of transforming growth factor-beta (TGFB1) signaling	[52]

FC, fold change.

*Adjusted P-value (Benjamini-Hochberg multiple testing correction).

**Table 3:** Overlap between the 17 differentially expressed genes observed in our study with other gene expression studies.

	Mean log_2_(fold-change)					
Gene symbol	Lockstone et al.[8]	Li et al. [9] a	Li et al. [9] b	Li et al. [9] c	Letourneau et al.[11]	Current study
*BCL2*	0.01	0.64	0.76	0.35	0.04	−0.91
*BCL2L1*	0.15	−1.91	0.25	1.27	−0.80	0.53
*CCL3*	NA	NA	NA	NA	2.29	−1.70
*CCL3*/*CCL3L1*/*CCL3L3*	−0.38	NA	NA	NA	NA	NA
*CCR2*	−0.01	0.26	0.10	0.48	−0.99	1.32
*CCR5*	−0.06	NA	NA	NA	−1.84	0.43
*CCR7*	−0.17	0.18	0	−0.33	0.44	−1.20
*CD19*	−0.14	0.03	0.12	−0.71	−0.08	−0.98
*CD28*	−0.08	0.47	0.29	−0.14	−0.40	−0.78
*CD40*	−0.11	−0.33	−0.01	−0.24	0.41	−0.44
*CD40LG*	−0.06	0.25	0.21	−0.23	−0.87	−0.93
*CD80*	0.02	0.27	0.04	−0.51	0.15	−0.94
*EDN1*	0.37	−0.07	−0.07	−0.28	−0.01	−1.78
*IKBKB*	0.12	0.40	0.45	0.20	−0.13	−0.70
*IL10*	−0.04	−0.05	0.33	0.33	0.46	1
*IL6*	−0.47	0.04	−0.33	−0.33	−1.80	−1.72
*IL6*	−0.47	−0.18	−0.17	−0.06	−1.80	−1.72
*NOS2*	−0.42	−0.26	0.03	−0.25	−0.82	−1.13
*SKI*	0.06	0.03	−0.06	0.08	−0.42	−0.46

a Neonate group (age: 3 days to 38 days).

b Child group (age:1 year to 13 years).

c Neonate and child group.

NA  =  not available.

## Discussion

Individuals with DS have increased susceptibility to infections, hematological malignancies and are more likely to develop certain autoimmune disorders compared with non-DS individuals [3]. In this study, we profiled the expression pattern of 92 genes in peripheral blood of two different groups, children with DS and controls, to identify differentially expressed genes that might be of pathogenetic importance for the development of the immunological alterations observed in individuals with DS. A set of 17 differentially expressed genes involved in immune and inflammatory pathways was identified including 13 genes (*BCL2*, *CCL3*, *CCR7*, *CD19*, *CD28*, *CD40, CD40LG*, *CD80*, *EDN1*, *IKBKB*, *IL6, NOS2* and *SKI*) significantly down-regulated and four genes (*BCL2L1*, *CCR2*, *CCR5* and *IL10*) significantly up-regulated in children with DS.

In our DS population we found a decreased number of typical lymphocytes and leukocytes and no alteration in the number of neutrophils, basophils, monocytes and eosinophils compared with controls. The reduced number of typical lymphocytes and leukocytes observed in our DS group is in agreement with prior literature data [21]–[23]. Changes in the lymphocyte number have been hypothesized to be due to thymus dysfunction and B and T cell apoptosis [24], [25]. All these findings suggest that the alterations of such parameters are the core of the immune system problem observed in individuals with DS. On the other hand, as pointed out by Kusters and collegues [26], children with congenital heart disease that go through cardiac surgery with (partial) thymectomy end up with permanent changes in T lymphocyte numbers however, in contrast with individuals with DS, their frequency of infections and autoimmune diseases is not increased. Thus, the underlying mechanisms for the immune dysfunction in DS remain unclear and have been considered to be associated with the gene dosage imbalance caused by the presence of an additional chromosome 21 (HSA21). The different ratio of lymphocytes and leukocytes to myeloid cells in individuals with DS versus controls observed in our study may lead one to argue that the reduction in leukocytes and lymphocytes is the responsible for the differentially expressed genes observed in our study. Although we cannot completely rule out this assumption, when we look at genes expressed in leukocytes and lymphocytes, such as *CCR2* and *CCR5*, one would expect them to be down-regulated in our DS group according to the difference in the ratio of leukocytes and lymphocytes to other white blood cells, which is not true.

Gene expression profiling studies using hybridization-based methodologies, such as microarray and PCR-based assays, offer a promising approach to understanding the complex network of gene changes that are associated with complex traits such immune impairment. Previous genome-wide expression studies have successfully applied microarray technology to investigate the transcriptional consequences of trisomy 21 in several different types of biomaterials such as leukocytes [7], adult brain tissue [8], PBMCs [9], lymphoblastoid cell lines [10] and fetal fibroblasts [11]. We decided to use PBMCs, a reservoir to collect information from various physiologic processes once it permeates almost all tissues and cells, therefore it is a potentially the best biomaterial for sampling for overall immune function and health.

We identified 17 immune-related genes differentially expressed in our group of individuals with DS. The main functions of the differentially expressed genes are summarized in Table 2. Confirming data in the literature, the reduced expression of *BCL2* observed in our study was previously reported in DS leukocytes in comparison to normal controls [7]. This antiapoptotic gene is essential for the survival of mature lymphocytes and memory B cells [27], [28]. Also, the overexpression of *IL10* in our DS group was also previously reported [29]. *IL10* overexpression can result in exacerbated inflammatory response leading to the development of hyper inflammatory disorders such as autoimmune diseases [30], [31], a well known phenomena observed in individuals with DS.

In order to further investigate the findings in our study, we evaluated the degree of overlap between our data and that from three other gene expression studies [8], [9], [11] for the 17 differentially expressed genes observed in our study (Table 3). Gene expression values of the 17 genes of each study had little overlap. The genes *CD40LG*, *Il6* and *NOS2* where down-regulated in our study and in study by Letourneau et al. [11], and the gene *CD19* was found significant down-regulated in our study and in Li et al. [9] when neonate and child groups were combined, a finding that confirms previous studies of lymphocyte subpopulations in fetuses [32] and in children with DS [33]. Little overlap between the individual genes identified in our study and the literature is not surprising. There are several possible reasons for this discordance notably differences in cell types used for analysis at different developmental stages and small sample size. Moreover, there are many experimental factors such as array platform, tissue handling, RNA isolation method and hybridization procedure which are likely to contribute to the observed divergence.

A strength of this study that make it different from previous gene expression studies is that we attempted to identify a selected pathway associated to a specific DS feature, the immunological impairment. While others have applied microarray analysis to perform global gene expression and then chose one or more genes for further investigation, we decided to investigate a selected number of genes associated to the specific immunological alteration observed in DS. For that matter, some confounding factors including medication ingestion, personal history of chronic infection, and clinical manifestations suggestive of acute infection were controlled. Also, the majority of the previous transcriptome studies were performed using microarrays methods. Despite a high level of agreement between qPCR and microarrays, it has been demonstrated that microarrays tend to have a low dynamic range, which could lead to under-representation of changes in gene expression, whereas qPCR has a higher dynamic range [34]. Our observations are clearly limited by the small number of individuals analyzed; a larger study population would provide a higher statistical power. Nevertheless, our findings identify a number of genes with relevant functions in immune cells that are dysregulated in DS children; in any case it remains of interest to investigate the expression pattern of these genes at the protein. The expectation is that the identification of such dosage imbalance may provide insights to identify promising candidate genes underlying DS phenotype and thus bring more effective therapy that will lead to future advances for a safer and more targeted therapeutic approach in the treatment of individuals with DS for everyday clinical care. It is also important to mention that all the genes observed with altered expression in this study are located on chromosomes other than HSA21, what gives further evidence to the theory that secondary transcriptional changes throughout the genome occur as a result of trisomy 21.

## Supporting Information

Table S1 Immune genes contained in the TaqMan Array Human Immune, Fast 96-Well Plate (Applied Biosystems, Carlsbad, California, USA).
(DOC)
