# Supplementary material for: Altered Expression of Immune-Related Genes in Children with Down Syndrome
Source: PLoS One. 2014 Sep 15;9(9):e107218. doi: 10.1371/journal.pone.0107218 (PMC4164533; doi:10.1371/journal.pone.0107218)
Supplement: Table S1 — Immune genes contained in the TaqMan Array Human Immune, Fast 96-Well Plate (Applied Biosystems, Carlsbad, California, USA). (DOC) [file pone.0107218.s001.doc]

**Table S1.** Immune genes contained in the TaqMan Array Human Immune, Fast 96-Well Plate (Applied Biosystems, Carlsbad, California, USA).

| **Well Position** | **Assay ID** | **Gene Symbol** |
| --- | --- | --- |
| **A01** | Hs99999901_s1 | *18S*^a^ |
| **A02** | Hs99999905_m1 | *GAPDH*^a^ |
| **A03** | Hs99999909_m1 | *HPRT1*^a^ |
| **A04** | Hs99999908_m1 | *GUSB*^a^ |
| **A05** | Hs00174092_m1 | *IL1A* |
| **A06** | Hs00174097_m1 | *IL1B* |
| **A07** | Hs00174114_m1 | *IL2* |
| **A08** | Hs00174117_m1 | *IL3* |
| **A09** | Hs00174122_m1 | *IL4* |
| **A10** | Hs00174200_m1 | *IL5* |
| **A11** | Hs00174131_m1 | *IL6* |
| **A12** | Hs00174202_m1 | *IL7* |
| **B01** | Hs00174103_m1 | *IL8* |
| **B02** | Hs00174125_m1 | *IL9* |
| **B03** | Hs00174086_m1 | *IL10* |
| **B04** | Hs00168405_m1 | *IL12A* |
| **B05** | Hs00233688_m1 | *IL12B* |
| **B06** | Hs00174379_m1 | *IL13* |
| **B07** | Hs00174106_m1 | *IL15* |
| **B08** | Hs00174383_m1 | *IL17A* |
| **B09** | Hs00155517_m1 | *IL18* |
| **B10** | Hs00234142_m1 | *CCL3* |
| **B11** | Hs00171149_m1 | *CCL19* |
| **B12** | Hs00234140_m1 | *CCL2* |
| **C01** | Hs00174575_m1 | *CCL5* |
| **C02** | Hs00174150_m1 | *CCR2* |
| **C03** | Hs99999919_m1 | *CCR4* |
| **C04** | Hs00152917_m1 | *CCR5* |
| **C05** | Hs00171054_m1 | *CCR7* |
| **C06** | Hs00171041_m1 | *CXCR3* |
| **C07** | Hs00171042_m1 | *CXCL10* |
| **C08** | Hs00171138_m1 | *CXCL11* |
| **C09** | Hs00174164_m1 | *CSF1* |
| **C10** | Hs00171266_m1 | *CSF2* |
| **C11** | Hs00357085_g1 | *CSF3* |
| **C12** | Hs00234174_m1 | *STAT3* |
| **D01** | Hs00174517_m1 | *NFKB2* |
| **D02** | Hs00233284_m1 | *IKBKB* |
| **D03** | Hs00167894_m1 | *CD3E* |
| **D04** | Hs00181217_m1 | *CD4* |
| **D05** | Hs00233520_m1 | *CD8A* |
| **D06** | Hs00174333_m1 | *CD19* |
| **D07** | Hs00166229_m1 | *IL2RA* |
| **D08** | Hs00174796_m1 | *CD28* |
| **D09** | Hs00233552_m1 | *CD38* |
| **D10** | Hs00374176_m1 | *CD40* |
| **D11** | Hs00365634_g1 | *PTPRC* |
| **D12** | Hs00154355_m1 | *CD68* |
| **E01** | Hs00175478_m1 | *CD80* |
| **E02** | Hs00199349_m1 | *CD86* |
| **E03** | Hs00175480_m1 | *CTLA4* |
| **E04** | Hs00163934_m1 | *CD40LG* |
| **E05** | Hs00219575_m1 | *HLA-DRA* |
| **E06** | Hs99999917_m1 | *HLA-DRB1* |
| **E07** | Hs00203436_m1 | *TBX21* |
| **E08** | Hs00188346_m1 | *TNFRSF18* |
| **E09** | Hs00359999_m1 | *ICOS* |
| **E10** | Hs00167248_m1 | *NOS2* |
| **E11** | Hs00153350_m1 | *BCL2* |
| **E12** | Hs00169141_m1 | *BCL2L1* |
| **F01** | Hs00180269_m1 | *BAX* |
| **F02** | Hs00164932_m1 | *ICAM1* |
| **F03** | Hs00174583_m1 | *SELP* |
| **F04** | Hs00174057_m1 | *SELE* |
| **F05** | Hs00157965_m1 | *HMOX1* |
| **F06** | Hs00153133_m1 | *PTGS2* |
| **F07** | Hs00189742_m1 | *LRP2* |
| **F08** | Hs00167927_m1 | *CYP1A2* |
| **F09** | Hs00167982_m1 | *CYP7A1* |
| **F10** | Hs00174143_m1 | *IFNG* |
| **F11** | Hs00169473_m1 | *PRF1* |
| **F12** | Hs00188051_m1 | *GZMB* |
| **G01** | Hs00246266_m1 | *GNLY* |
| **G02** | Hs00163653_m1 | *FAS* |
| **G03** | Hs00181225_m1 | *FASLG* |
| **G04** | Hs00171257_m1 | *TGFB1* |
| **G05** | Hs00232222_m1 | *SMAD3* |
| **G06** | Hs00178696_m1 | *SMAD7* |
| **G07** | Hs00161707_m1 | *SKI* |
| **G08** | Hs00365052_m1 | *FN1* |
| **G09** | Hs00163811_m1 | *C3* |
| **G10** | Hs00174128_m1 | *TNF* |
| **G11** | Hs00236874_m1 | *LTA* |
| **G12** | Hs00174179_m1 | *ACE* |
| **H01** | Hs00173626_m1 | *VEGFA* |
| **H02** | Hs00156373_m1 | *CD34* |
| **H03** | Hs00241341_m1 | *AGTR1* |
| **H04** | Hs00169126_m1 | *AGTR2* |
| **H05** | Hs00174961_m1 | *EDN1* |
| **H06** | Hs00171455_m1 | *LIF* |
| **H07** | Hs00209771_m1 | *LY96* |
| **H08** | Hs00236988_g1 | *MIF* |
| **H09** | Hs00190046_m1 | *NFATC3* |
| **H10** | Hs00190037_m1 | *NFATC4* |
| **H11** | Hs00236998_m1 | *PF4* |
| **H12** | Hs00374292_m1 | *SYK* |

^a^ reference genes.
